# Supplementary material for: Notch dimerization and gene dosage are important for normal heart development, intestinal stem cell maintenance, and splenic marginal zone B-cell homeostasis during mite infestation
Source: PLoS Biol. 2020 Oct 5;18(10):e3000850. doi: 10.1371/journal.pbio.3000850 (PMC7561103; doi:10.1371/journal.pbio.3000850)
Supplement: S1 Fig — (A-B) EMSA data for purified proteins binding to probes containing 2 CSL (A) or SPS (B) sites. The number of balls marks the occupancy of sites; arrows below indicate orientation of binding sites in probes. (C-D) The average sites filled were plotted against RBPj concentrations to calculate the cooperativity factor “C” on CSL (C) and SPS (D) probes. (E) Relative expression of Notch target genes in mK4 cells overexpressing either N1ΔE or N1RAΔE as determined by RNA Seq (all replicates are shown, values ranked per row. Green arrows indicate targets elevated more in N1RAΔE, red arrows indicate targets repressed in in N1RAΔE relative to control. (F) Luciferase activation assays were used to analyze the homo- and heterodimerization properties of Notch1 and Notch2 on a dimer-dependent reporter gene (Hes5-Luciferase) and a dimer-agnostic reporters (Hey2-Luciferase). As previously shown [38], the dimer interface involves 3 amino acids, the Arg (R) mutated in this study, as well as the positively charged Lys (K1935, Notch1) and a negatively charged Glu (E1939, Notch1, or Asp [D1899] at the equivalent position in Notch2) that form salt bridges between NICD molecules. Dimer formation is critical for Notch-dependent activation of the Hes5 reporter, and mutating any of these amino acids abrogates activation. Notably, by co-expressing 2 Notch protein with complementary mutations, one in which K1935 was changed to E (N1KE), and a second in which D1899 was changed to K (N2DK) leads to strong enhancement in Luciferase expression, explained by achieving a more favorable conformation of the complementing mutant dimer. Note synergistic complementation between N1KE and N2DK but not between N1KE and N2KD, providing strong evidence of a cooperative heterodimer. Significance tested by a Student t-test. CSL, CBF1/Suppressor of Hairless/LAG-1; EMSA, in electrophoretic mobility shift assay; NICD, notch intracellular domain; N1RAΔE, extracellular domain deleted Notch1 Arg1974Ala mutant; RPBj, [file pbio.3000850.s001.pdf]

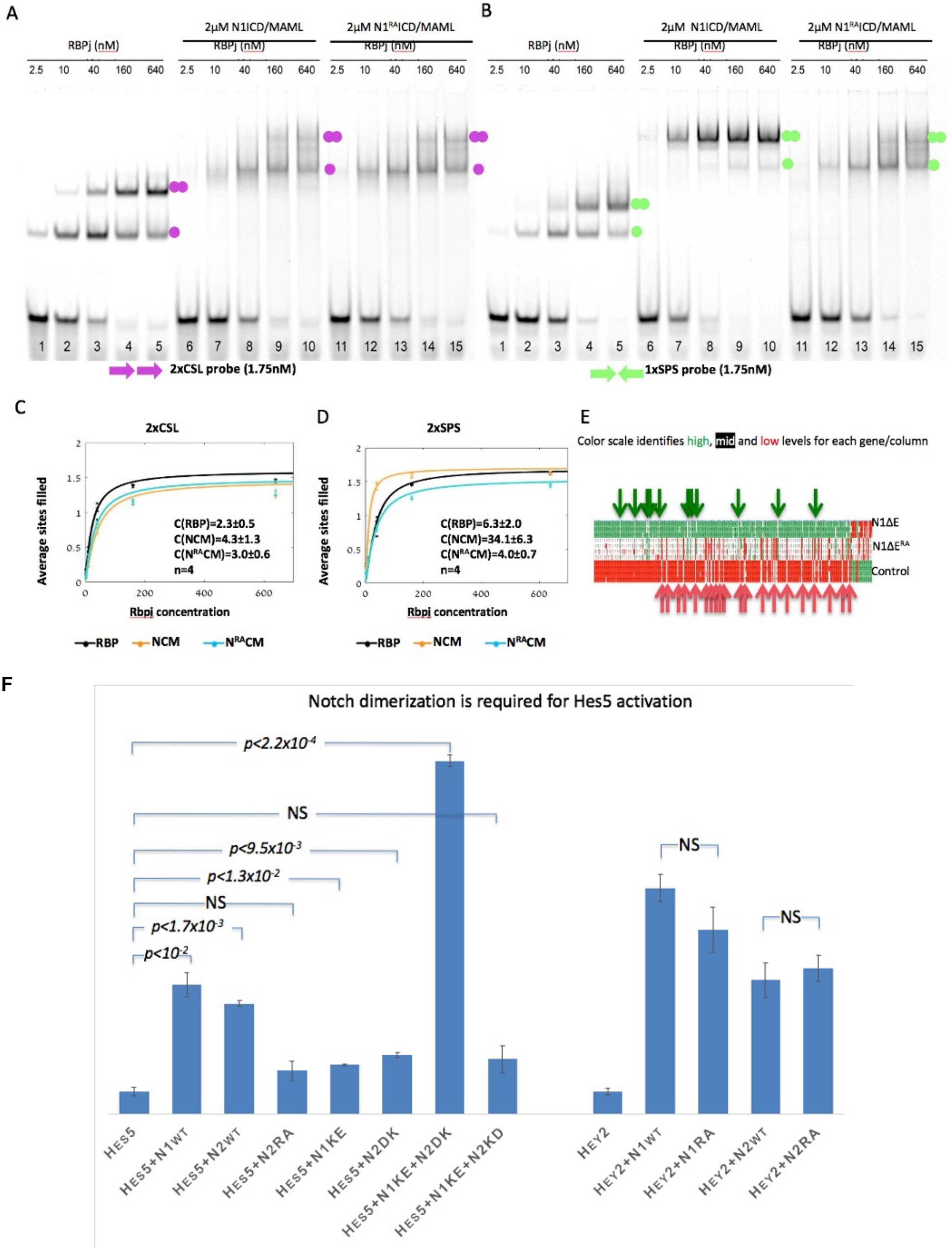
A
RBPj (nM)
2µM N1ICD/MAML
2µM N1^RA ICD/MAML
2.5 10 40 160 640
1 2 3 4 5 6 7 8 9 10 11 12 13 14 15
2xCSL probe (1.75nM)
B
1xSPS probe (1.75nM)
C
2xCSL
Average sites filled
Rbpj concentration
C(RBP)=2.3±0.5
C(NCM)=4.3±1.3
C(N^RA CM)=3.0±0.6
n=4
RBP NCM N^RA CM
D
2xSPS
C(RBP)=6.3±2.0
C(NCM)=34.1±6.3
C(N^RA CM)=4.0±0.7
n=4
E
Color scale identifies high, mid and low levels for each gene/column
N1ΔE
N1ΔE^RA
Control
F
Notch dimerization is required for Hes5 activation
p<2.2x10-4
NS
p<9.5x10-3
p<1.3x10-2
NS
p<1.7x10-3
p<10-2
NS
NS
Hes5
Hes5+N1WT
Hes5+N2WT
Hes5+N2RA
Hes5+N1KE
Hes5+N2DK
Hes5+N1KE+N2DK
Hes5+N1KE+N2KD
Hey2
Hey2+N1WT
Hey2+N1RA
Hey2+N2WT
Hey2+N2RA

S1 Fig. Loss of Notch1 dimerization suppresses target gene expression in MK4 cells (supporting Fig 1, see S1 Data for raw data). (A-B) EMSA data for purified proteins binding to probes containing two CSL (A) or SPS (B) sites. The number of balls marks the occupancy of sites, arrows below indicate orientation of binding sites in probes. (C-D) The average sites filled were plotted against RBPj concentrations to calculate the cooperativity factor "C" on CSL (C) and SPS (D) probes. (E) Relative expression of Notch target genes in mK4 cells overexpressing either N1ΔE or $N1^{RA}ΔE$ as determined by RNA Seq (all replicates are shown, values ranked per row. Green arrows indicate targets elevated more in $N1^{RA}ΔE$, red arrows indicate targets repressed in in $N1^{RA}ΔE$ relative to control. (F) Luciferase activation assays were used to analyze the homo and heterodimerization properties of Notch1 and Notch2 on a dimer-dependent reporter gene (Hes5-Luciferase) and a dimer-agnostic reporters (Hey2-Luciferase). As previously shown (Arnett et al., 2010), the dimer interface involves three amino acids, the Arg (R) mutated in this study, as well as the positively charged Lys (K1935, Notch1) and a negatively charged Glu (E1939, Notch1 or Asp (D1899) at the equivalent position in Notch2) that form salt bridges between NICD molecules. Dimer formation is critical for Notch-dependent activation of the Hes5 reporter, and mutating any of these amino acids abrogates activation. Notably, by co-expressing two Notch protein with complementary mutations, one in which K1935 was changed to E (N1KE), and a second in which D1899 was changed to K (N2DK) leads to strong enhancement in Luciferase expression, explained by achieving a more favorable conformation of the complementing mutant dimer. Note synergistic complementation between N1KE and N2DK, but not between N1KE and N2KD, providing strong evidence of a cooperative heterodimer. Significance tested by a student t test.
