# Supplementary material for: Notch dimerization and gene dosage are important for normal heart development, intestinal stem cell maintenance, and splenic marginal zone B-cell homeostasis during mite infestation
Source: PLoS Biol. 2020 Oct 5;18(10):e3000850. doi: 10.1371/journal.pbio.3000850 (PMC7561103; doi:10.1371/journal.pbio.3000850)
Supplement: S2 Fig — Thymi and spleens were isolated from wt, N1+/RA, or N1RA/RA mice, and the T-cell compartment was analyzed. (A) The absolute number of thymic single- and double-positive T cells (left) and developing T cells (right) was assessed by flow cytometry. (A’) The average number of cells in the thymus (left) and average thymic weight (right) are shown. (B) The percentage (left) and absolute number (right) of T-cell subsets in the spleen were assessed by flow cytometry. (B’) The average number of splenocytes (left) and average spleen weight (right) are shown. (n = 3–6 mice per genotype; error bars = +/- SEM). N1+/RA, Notch1 Arg1974Ala heterozygote; N1RA/RA, Notch1 Arg1974Ala homozygote; wt, wild-type. (PDF) [file pbio.3000850.s002.pdf]

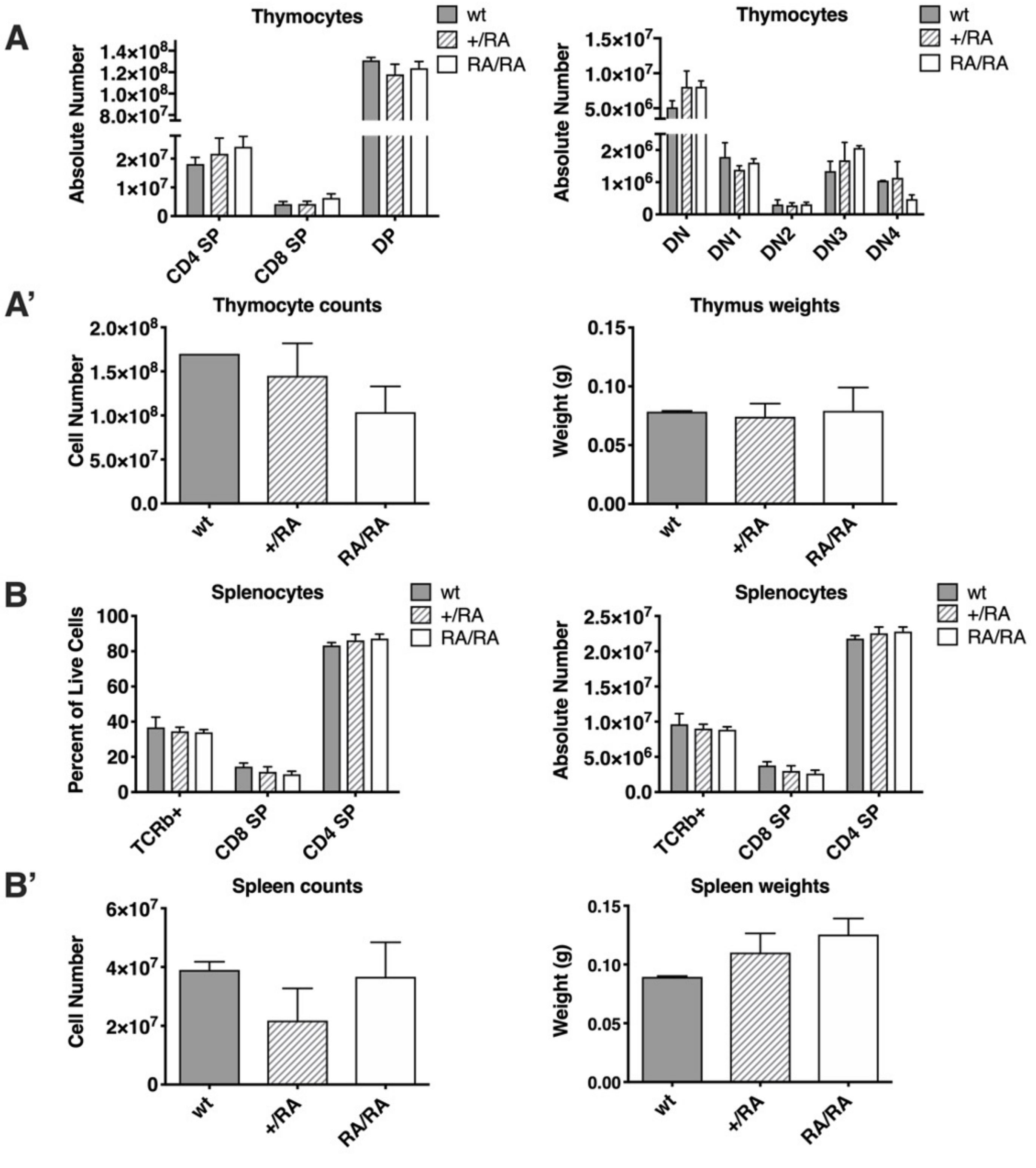

S2 Fig. Normal development of T cell compartments in *N1*$^{RA/RA}$ mice (supporting Fig 1, see S1 Data for raw data). Thymi and spleens were isolated from *wt*, *N1*$^{+/RA}$, or *N1*$^{RA/RA}$ mice, and the T cell compartment was analyzed. (**A**) The absolute number of thymic single- and double-positive T cells (left) and developing T cells (right) was assessed by flow cytometry. (**A’**) The average number of cells in the thymus (left) and average thymic weight (right) are shown. (**B**) The percentage (left) and absolute number (right) of T cell subsets in the spleen were assessed by flow cytometry. (**B’**) The average number of splenocytes (left) and average spleen weight (right) are shown. (n=3-6 mice per genotype; error bars = +/- SEM).
