# Supplementary material for: Notch dimerization and gene dosage are important for normal heart development, intestinal stem cell maintenance, and splenic marginal zone B-cell homeostasis during mite infestation
Source: PLoS Biol. 2020 Oct 5;18(10):e3000850. doi: 10.1371/journal.pbio.3000850 (PMC7561103; doi:10.1371/journal.pbio.3000850)
Supplement: S3 Fig — Sections of P0 and P1 intestines from animals born to fur mite–infested dams (A-H’) and born after fur mite eradication (I-J’) were stained for Ki67 (A-D’, I, and I’) and phospho-H3 (E-H’, J, and J’) to assess for proliferation in the crypts. N1+/-;N2+/- are indistinguishable from wt (A-B’, E-F’), whereas proliferation is significantly reduced in N1+/-;N2RA/RA and N1RA/-;N2RA/- crypts (C-D’, G-H’). Proliferation in N1RA/-;N2RA/- is still decreased compared with heterozygous littermates after fur mite eradication (I-J’). N1+/-;N2+/-, Notch1/Notch2 RA hemizygous; N2RA/RA, Notch2 RA homozygous; P0-1, postnatal day 0–1; RA, Arg (N1R1974/N2R1934) to Ala substitution; wt, wild-type. (PDF) [file pbio.3000850.s003.pdf]

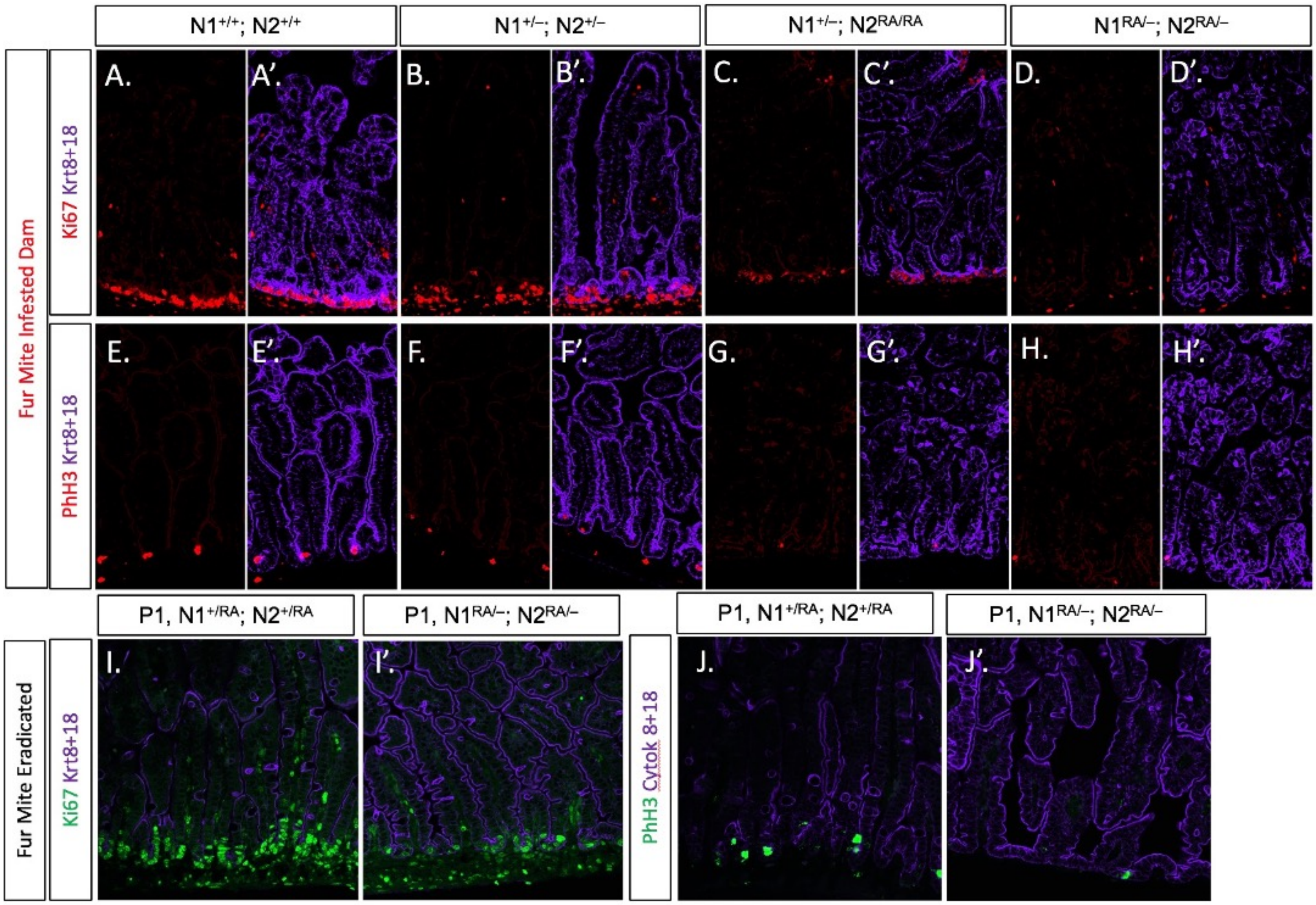


**S3 Fig. Proliferation potential in wild type vs mutant intestines with or without fur mite exposure (supporting Fig 4).** Sections of P0 and P1 intestines from animals born to fur mite infested dams (A-H') and born after fur mite eradication (I-J') were stained for Ki67 (A-D', I and I') and phosph-H3 (E-H', J and J') to assess for proliferation in the crypts. *$N1^{+/-};N2^{+/-}$* are indistinguishable from *wt* (A-B', E-F'), while proliferation is significantly reduced in *$N1^{+/-};N2^{RA/RA}$* and *$N1^{RA}/-;N2^{RA/-}$* crypts (C-D', G-H'). Proliferation in *$N1^{RA/-};N2^{RA/-}$* is still decreased compared to heterozygous littermates after fur mite eradication (I-J').
