# Supplementary material for: Notch dimerization and gene dosage are important for normal heart development, intestinal stem cell maintenance, and splenic marginal zone B-cell homeostasis during mite infestation
Source: PLoS Biol. 2020 Oct 5;18(10):e3000850. doi: 10.1371/journal.pbio.3000850 (PMC7561103; doi:10.1371/journal.pbio.3000850)
Supplement: S4 Fig — Isolated MZB from N2RA/RA and wt littermates were cultured and stimulated with LPS. Both genotypes proliferate after stimulation (A). Sections of N2RA/RA and wt spleens were stained with Ki67 and phosphor-H3 to detect proliferation and Caspase3 for apoptosis (B). Proliferation in germinative centers were detected in dimer-deficient mice but not in wt. Apoptosis was not altered. LPS, lipopolysaccharide; MZB, marginal zone B-cell; Notch2 RA homozygous; RA, Arg (N1R1974/N2R1934) to Ala substitution; wt, wild-type. (PDF) [file pbio.3000850.s004.pdf]

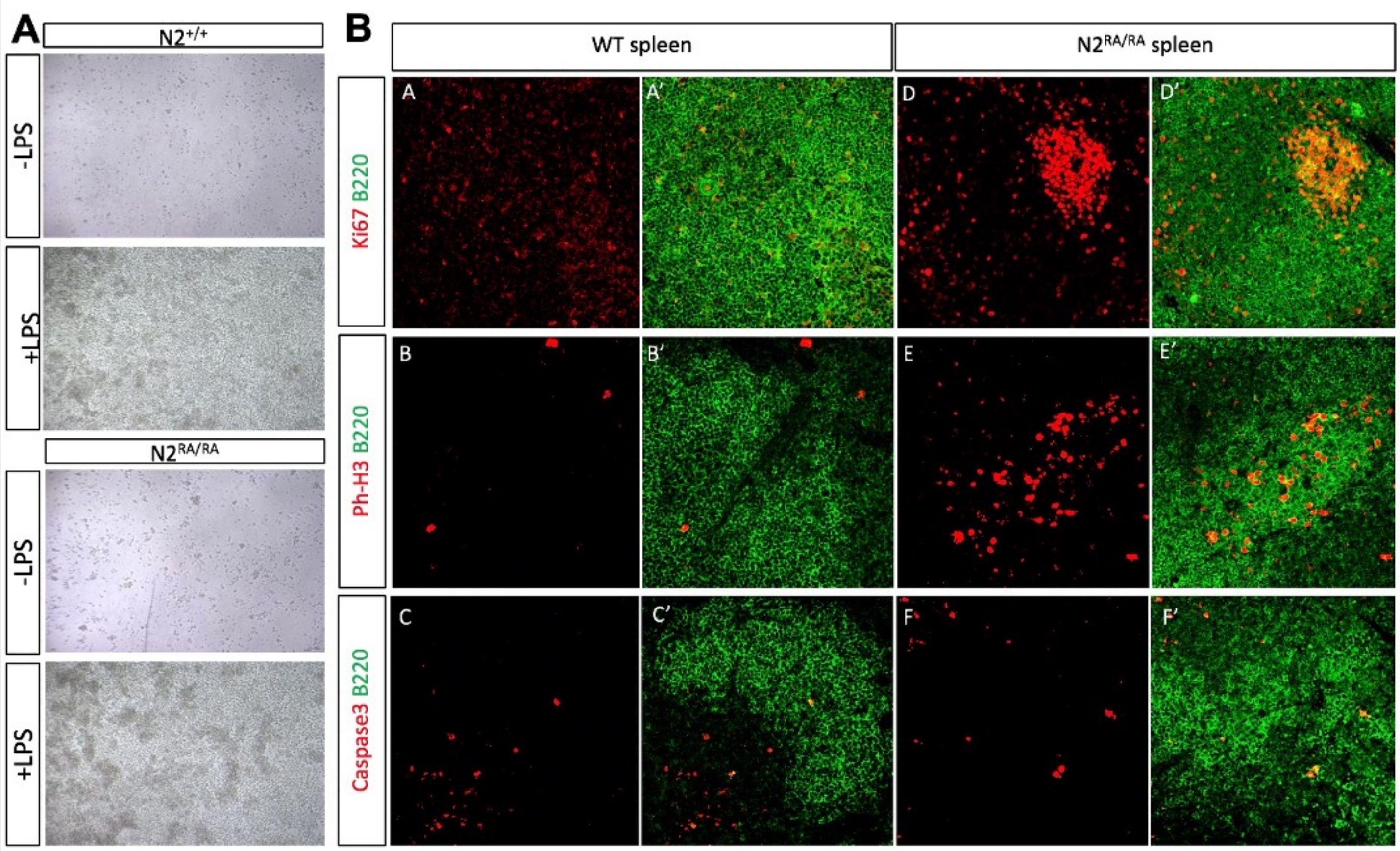

S4 Fig. Like wildtype MZB cells, *N2$^{RA/RA}$* MZB cells have a robust proliferative response upon LPS stimulation and *N2$^{RA/RA}$* spleen display germinative centers (supporting Fig 5). Isolated MZB from *N2$^{RA/RA}$* and *wt* littermates were cultured and stimulated with LPS. Both genotypes proliferate after stimulation (A). Sections of *N2$^{RA/RA}$* and *wt* spleens were stained with Ki67 and phospho-H3 to detect proliferation and Caspase3 for apoptosis (B). Proliferation in germinative centers were detected in dimer deficient mice, but not in *wt*. Apoptosis was not altered.
