# Supplementary material for: Notch dimerization and gene dosage are important for normal heart development, intestinal stem cell maintenance, and splenic marginal zone B-cell homeostasis during mite infestation
Source: PLoS Biol. 2020 Oct 5;18(10):e3000850. doi: 10.1371/journal.pbio.3000850 (PMC7561103; doi:10.1371/journal.pbio.3000850)
Supplement: S5 Fig — In the absence of pathogens; N1+/RA;N2RA/RA enlarged spleens and lymph nodes of mite-infested mice have a high proliferative and mitotic index (S6 Fig, see S1 Data for raw data). (A). In the absence of fur mites, an increase in spleen size with aging was observed in N2RA/RA mice (RA), but not in mice with other genotypes (+) housed in the same colony (B, D). The enlarged spleens and lymph nodes from aged N1+/RA;N2RA/RA mice showed increased staining for Ki67 and phosphor-H3 indicating proliferation Apoptosis was slightly increased in enlarged spleens as shown by Caspase3 stain (C). (E) Low magnification of spleens from aged N2RA/RA and N1+/RA;N2RA/RA mice infested with fur mites show expansion of white pulp. N1+/RA;N2RA/RA, Notch1 RA heterozygote, Notch2 RA homozygous; RA, Arg (N1R1974/N2R1934) to Ala substitution. (PDF) [file pbio.3000850.s005.pdf]

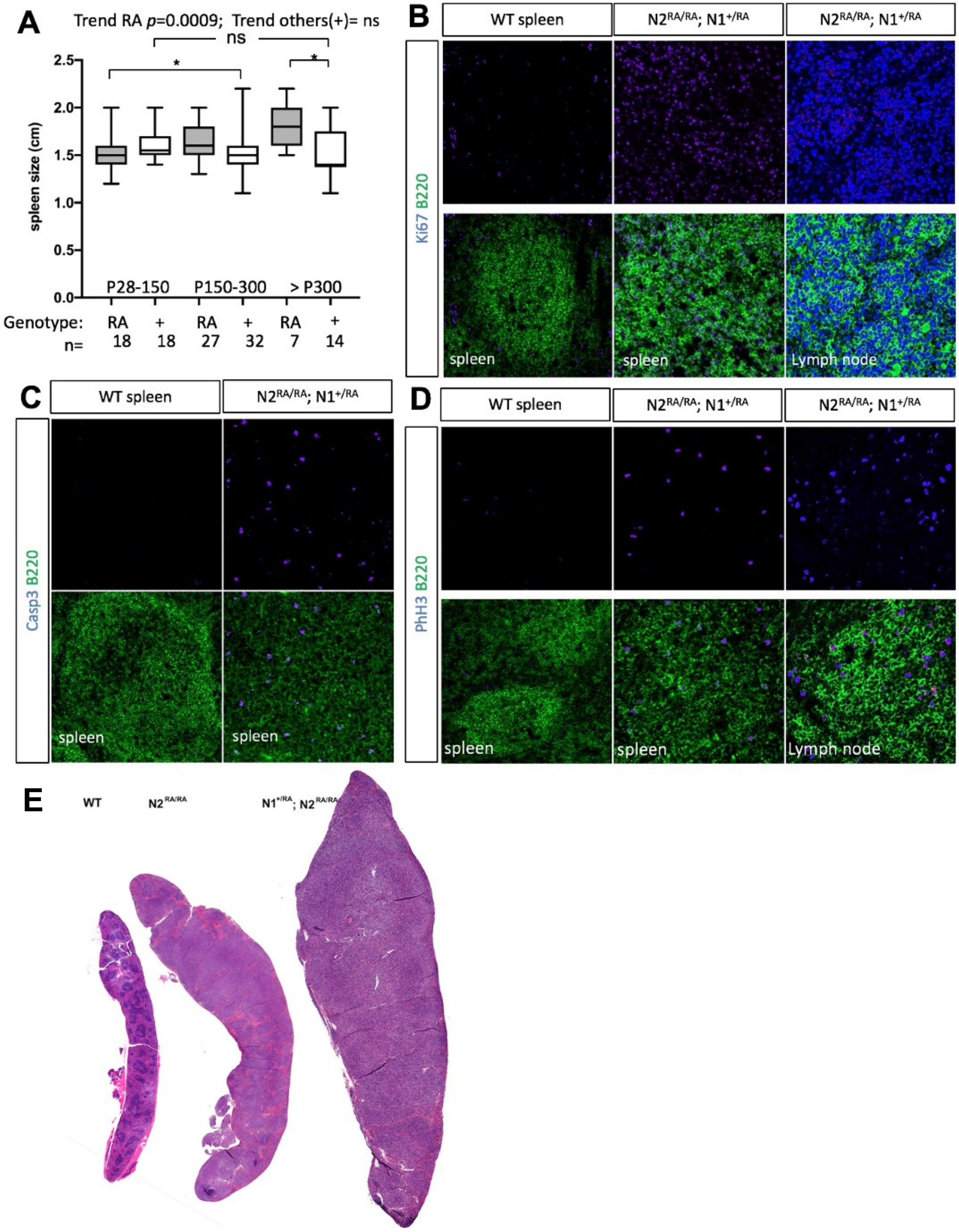

A
Trend RA p=0.0009; Trend others(+)= ns
ns
*
*
spleen size (cm)
2.5
2.0
1.5
1.0
0.5
0.0
P28-150
P150-300
> P300
Genotype: RA + RA + RA +
n= 18 18 27 32 7 14
B
WT spleen
N2RA/RA; N1+/RA
N2RA/RA; N1+/RA
Ki67 B220
spleen
spleen
Lymph node
C
WT spleen
N2RA/RA; N1+/RA
Casp3 B220
spleen
spleen
D
WT spleen
N2RA/RA; N1+/RA
N2RA/RA; N1+/RA
PhH3 B220
spleen
spleen
Lymph node
E
WT
N2RA/RA
N1+/RA; N2RA/RA

S5 Fig. Spleen size as a function of age In the absence of pathogens; *N1*$^{+/RA}$*;N2*$^{RA/RA}$ enlarged spleens and lymph nodes of mite infested mice have a high proliferative and mitotic index (supporting Fig 6, see S1 Data for raw data). (A). In the absence of fur mites, an increase in spleen size with aging was observed in *N2*$^{RA/RA}$ mice (RA), but not in mice with other genotypes (+) housed in the same colony. (B, D). The enlarged spleens and lymph nodes from aged *N1*$^{+/RA}$*;N2*$^{RA/RA}$ mice showed increased staining for Ki67 and phosphor-H3 indicating proliferation Apoptosis was slightly increased in enlarged spleens as shown by Caspase3 stain (C). (E) Low magnification of spleens from aged *N2*$^{RA/RA}$ and *N1*$^{+/RA}$*;N2*$^{RA/RA}$ mice infested with fur mites show expansion of white pulp.
