# Supplementary material for: Notch dimerization and gene dosage are important for normal heart development, intestinal stem cell maintenance, and splenic marginal zone B-cell homeostasis during mite infestation
Source: PLoS Biol. 2020 Oct 5;18(10):e3000850. doi: 10.1371/journal.pbio.3000850 (PMC7561103; doi:10.1371/journal.pbio.3000850)
Supplement: S6 Fig — Western blot analysis of nuclear (N) and cytoplasmic (C) preparations of sorted MZB shows no difference in N2 stability between wt and N2RA/RA mice (A); immunoblotting of α-tubulin and histone-h3 confirms separation of cytoplasmic and nuclear fractions, respectively. Quantification of the nuclear N2ICD relative to cytoplasmic total N2 reveals no difference between wt and dimer-deficient N2ICD stability (B). MZB, marginal zone B-cell; NICD, Notch intracellular domain; wt, wild-type. (PDF) [file pbio.3000850.s006.pdf]

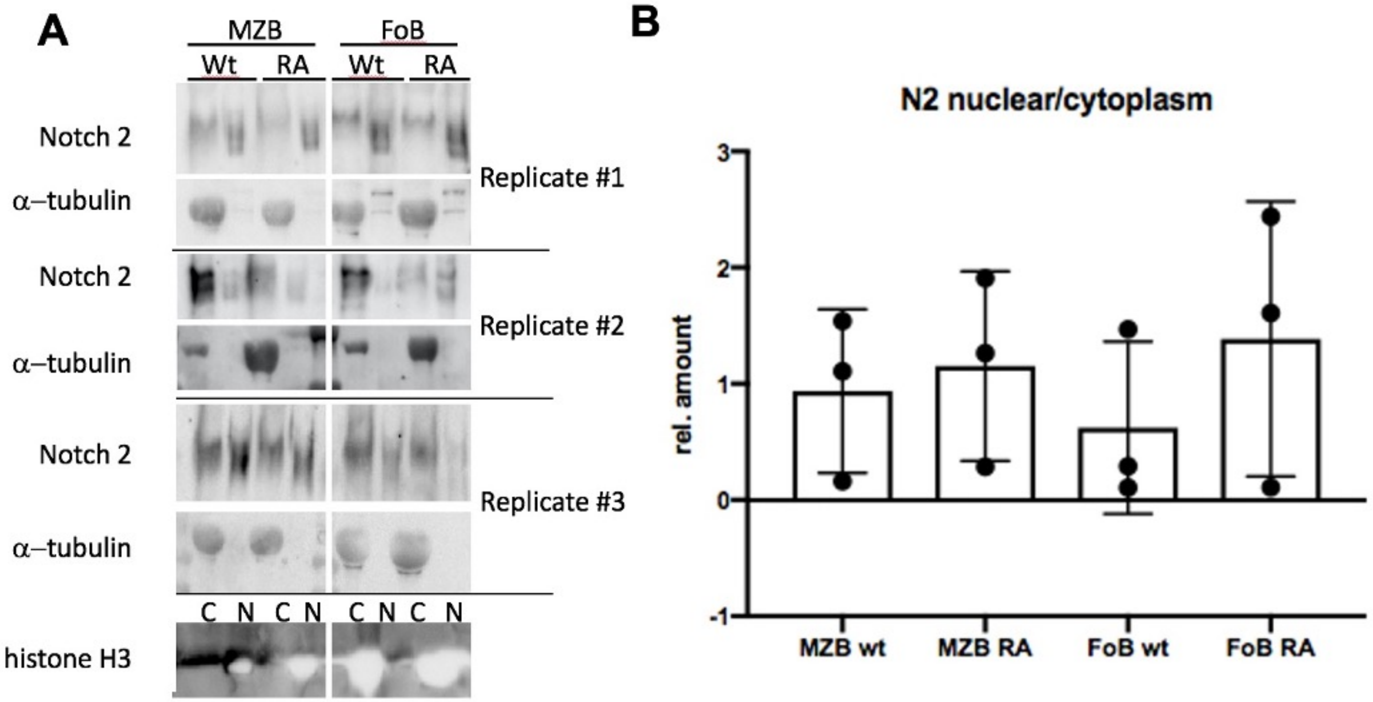

S6 Fig. Loss of NICD Dimerization does not stabilize the protein (supporting Fig 6; see S1 Data for raw data**).** Western blot analysis of nuclear (N) and cytoplasmic (C) preparations of sorted MZB shows no difference in N2 stability between *wt* and *N2*$^{RA/RA}$ mice (A); immunoblotting of α-tubulin and histone-h3 confirms separation of cytoplasmic and nuclear fractions, respectively. Quantification of the nuclear N2ICD relative to cytoplasmic total N2 reveals no difference between wt and dimer deficient N2ICD stability (B).
