# Supplementary material for: Notch dimerization and gene dosage are important for normal heart development, intestinal stem cell maintenance, and splenic marginal zone B-cell homeostasis during mite infestation
Source: PLoS Biol. 2020 Oct 5;18(10):e3000850. doi: 10.1371/journal.pbio.3000850 (PMC7561103; doi:10.1371/journal.pbio.3000850)
Supplement: S1 Table — N1, Notch1; N2, Notch2; RA, Arg (N1R1974/N2R1934) to Ala substitution; P, postnatal day. (PDF) [file pbio.3000850.s007.pdf]

S1 Table. Chi-squared analysis of pups (p0 and p1) born in of *N1*$^{RA/RA}$*;N2*$^{RA/RA}$ x *N1*$^{+/-}$*;N2*$^{+/-}$ cross in mite-free housing

		expected		*observed*		$\frac{(O-E)^2}{O}$
F1	*N1*$^{RA/-}$; *N2*$^{RA/-}$	25%	47.5	14	7.37%	23.63
	N1$^{RA/-}$; *N2*$^{+/RA}$	25%	47.5	47	24.74%	0.01
	N1$^{+/RA}$; *N2*$^{RA/-}$	25%	47.5	51	26.84%	0.26
	N1$^{+/RA}$; *N2*$^{+/RA}$	25%	47.5	78	41.05%	19.58
		100%	190	190	100%	43.47
Degree of freedom (# of genotypes -1)=3					CHISQ.DIST	***p*=10^{-9}**
