# Supplementary material for: Notch dimerization and gene dosage are important for normal heart development, intestinal stem cell maintenance, and splenic marginal zone B-cell homeostasis during mite infestation
Source: PLoS Biol. 2020 Oct 5;18(10):e3000850. doi: 10.1371/journal.pbio.3000850 (PMC7561103; doi:10.1371/journal.pbio.3000850)
Supplement: S2 Table — N1+/RA, Notch1 Arg1974Ala heterozygote. (PDF) [file pbio.3000850.s008.pdf]

S2 Table. Chi-squared analysis of male and female pups born in C57BL/6J *N1*$^{+/RA}$ x *N1*$^{+/RA}$ cross during fur mite infestation and following generations

**Males**		*expected*		*observed* (male/female)		$\frac{(O-E)^2}{O}$
F1	*N1*$^{RA/RA}$	25%	14.25	12	21.1%	0.36
	N1$^{RA/+}$	50%	28.5	33	57.9%	0.71
	N1$^{+/+}$	25%	14.25	12	21.1%	0.36
		100%	57	57	100%	1.42
Degree of freedom (# of genotypes -1)=2				CHISQ.DIST		***p*=0.49**

**Females**		*expected*		*observed* (male/female)		$\frac{(O-E)^2}{O}$
F1	*N1*$^{RA/RA}$	25%	12.5	3	6.0%	7.22
	N1$^{RA/+}$	50%	25	30	60.0%	1.00
	N1$^{+/+}$	25%	12.5	17	34.0%	1.62
		100%	50	50	100%	9.84
Degree of freedom (# of genotypes -1)=2				CHISQ.DIST		***p*=0.01**
