# Supplementary material for: Notch dimerization and gene dosage are important for normal heart development, intestinal stem cell maintenance, and splenic marginal zone B-cell homeostasis during mite infestation
Source: PLoS Biol. 2020 Oct 5;18(10):e3000850. doi: 10.1371/journal.pbio.3000850 (PMC7561103; doi:10.1371/journal.pbio.3000850)
Supplement: Attachment — Submitted filename: Response to Reviewers.docx [file pbio.3000850.s010.docx]

Reviewers’ comments

Rev. 1:

This manuscript is a deep, thorough, and fascinating exploration of the functional consequences of introducing single point substitutions into murine Notch1 and Notch2 that interfere with cooperative dimerization of Notch transcription complexes at head-to-head paired site elements (SPS sites). In isolation, neither dimer-deficient variant results in a developmental phenotype, nor does combining the two alleles in a double homozygote. Under environmental stress, however, N1 RA/RA, N2 RA/RA mice exhibit colonic barrier defects in a DSS colitis model. Analysis of N1 RA/-, N2 +*/RA mice shows partial penetrance of a ventricular septal defect that is more pronounced in N1 RA/*-, N2 RA/- mice, and fur-mite challenged N2 RA/RA mice show marginal zone B (MZB) cell expansion that can resemble MZB lymphoma in aged mice. Mechanistic studies to elucidate the origin of the paradoxical MZB gain-of-function phenotype in the N2 RA/RA mice suggests that selective loading of this N2 allele on

monomer-responsive genes accounts for this effect. This manuscript certainly warrants publication in PLOS Biology after a few minor concerns are addressed.

Comments:

1. The mite infestation appears to have been an unplanned stressor of the genetically engineered mice. Did the authors evaluate whether the mites preferentially colonized the mutant mice compared with the wild-type mice?

On 12/20/2017 sentinels in our colony tested positive for fur mites and the entire colony was treated, including the NotchRA mice, but infestation was not limited to just them. We have no data to suggest they were the only or the most infected, and the caregivers did not report any excessive grooming, itching, or scruffy fur that would have indicated a heavy parasite load. Moreover, we have reestablished an isolated cohort of RA and control animals, and no differences in parasite burden between RA and control animals were noticed in the newly infected mouse colony.

2. In figure 5E, the authors make the point that HDM-induced dermatitis elevated MZB numbers only in the N2 RA/RA mice but not in controls (such as LPS-treated mice). The data do, however, show the same trend in the permethrin and LPS-treated mice - it is just that the data are somewhat noisy and the trends in the other conditions don't reach statistical significance. Could it be that the study was underpowered? The authors might make note of the trend, without undermining the overall message of the figure panel or the study.

We have tested 21 (10+11) mice with mites and 22 (11+11) mice post-mites. For an alpha=0.05, beta =0.2, and power=0.8 nine animals were enough for a significant score with fur mites (allowing for unequal variance), but 212 animals/group would be needed in the post-fur mite to show this trend was significant based on two-sided two-sample t-tests allowing unequal variance. Based on the values we see with LPS, 41 animals/group would be needed to establish significance. A significant change with HDM was observed with 4 animals/group. We interpret this data to suggest that although LPS treatment trended to impact the RA MZB more than the WT MZB, it was less impactful than fur mites or HDM by an order of magnitude. We will amend the text to reflect this point.

3. For those readers who are not Notch cognoscenti, the authors should expand the text to clarify for the more general reader the split-dam methodology use to compare loading of Notch-RBPJ monomeric complexes and dimeric complexes (that have NICD-D/NICD-AM within the same complex in order to build a functional DAM molecule). They should also improve the quality of the figure legend (D/AM "haves" I think means D/AM "halves" - and the text could also be made clearer for a naïve reader).

Thanks for the helpful comments- we revised the text and the legend.

Rev. 2:

Kobia et al. described the in vivo roles of cooperative DNA binding of intracellular domains of Notch receptors in heart, intestinal epithelium, and spleen. By meticulous phenotypical analysis of sophisticated knock-in mouse models of Notch1 and Notch2, authors found that dimerization-dependent Notch signaling is essential for heart development, injury response of colonic stem cells, and homeostasis of marginal zone B-cell. The most intriguing finding is Notch cooperativity can be induced at a certain specific context such as mite infestation, which is not preferred in a normal physiological status.

Dimer-deficient mice generated in this study are wonderful model system to investigate the differential roles between the conventional NTCs and the cooperative NTCs on SPS in vivo. The most of analysis were focused on phenotypical differences in various combination of dimer-compatible and dimer-defective alleles with different Notch dosages. Those are still very invaluable and informative resources to comprehend the fine-tuning mechanisms of diverse outcomes of Notch pathways. However, the underlying molecular mechanisms (provided mostly by Figure 7, only differential gene expression by RNA-seq analysis were examined) are not quite sufficient to explain the complex phenotypes of N1RA and N2RA alleles. Dissection of dimer-dependent and dimer-independent gene regulation using specific cell types affected in animal models (e.g. marginal zone B cells or intestinal stem cells) will greatly improve the manuscript. Detail comments are described below.

Major comments

- Perhaps, one of the most intriguing question would be where dimer-compatible NTC and dimer-defective NTC regulate gene expression. Do they regulate differential enhancer element in normal physiology and inflammatory environment (mite infestation or 1% DDS treated)? ATAC-seq analysis can be one of evidences, but it showed minimal changes in chromatin status between WT and N2RA/RA mice. SplitDAM experiments in Figure 7F is quite interesting but were performed in non-physiological context and only showed Myb locus. CHIP-seq analysis of Notch2+/+ and Notch2RA/RA can be a feasible approach to address this question.

Unfortunately, ChIP will not be helpful in this case because monomeric Notch binds to SPS sites, but does so non-cooperatively (see Figure S1 in this paper). Thus, both Notch and NotchRA variants do ChIP the same sites. We published this in our Hass et al 2015 Dev cell paper as Supplemental Figure S4. I am including the relevant panel from this Figure below. We will address this in the text as well.

*Figure S4: ChIP-PCR analysis of dimer-deficient Notch. Both N1^wt^ and N1^R1974A^ ChIP the Nrarp -3kb, Nrarp -8kb, and Nrarp -12kb enhancers but not the Nrarp -6kb control region, underscoring the inability of ChIP to differentiate between sites bound by Notch monomers (-12kb site) and dimers (-3kb and -8kb sites).*

The SplitDAMID is the only tool that enabled analysis of dimer/non-dimer binding by comparing the Notch-RBP pair (dimer + monomer) to the Notch-Notch pair (dimer but not monomer). Below, I am attaching an image prepared for the reviewer showing differential marking of SPS containing enhancers at –3kb and –8kb but not to the non-SPS –12kb enhancer in the *Nrarp* regulatory region, analyzed in our previous publication (Figure 4 in (Hass et al., 2015)).

In the image above we show that RBP ChIP pools down all three enhancers (bottom lane, blue, the same region analyzed by ChIP-PCR in Figure S4, above). The GATC-flanked DNA fragments are shown on top as alternating white and yellow bars, and the RBP-bound fragment is framed in a dashed red box. Unfused D/AM halves(in gray) mark open chromatin poorly. Reconstituted DAM pairs methylate adjacent GATC sites within ~1600bp on either side of the ChIP peak, which allow recovery by DpnI digest and PCR. Small (<50bp) or large (>2kb) fragments are recovered poorly, which is why the SplitDamID tags are not always overlapping the ChIPed fragment. Note that N/RBPj and N/N pairs mark the same fragments near the –3 and –8 enhancers (both containing functional SPS as we show in Hass et al, 2015) but only N/RBPj recover the flanking tag next to the –12 enhancers (which lacks the SPS).

The cost of generating SpiltDamID animals and the time it will require may prove out of my reach… We will happily share all reagents to enable this experiment to any interested party.

- Where are inflammation-sensitive SPS sites contributing altered gene expression in dimer-defective animal models? How can those SPSs upregulate or downregulate Notch target genes? Providing few examples (eg. Myb, FoxM1, E2F1) would be very helpful to understand the potential molecular mechanism of differential Notch pathway outcomes.

We now display these sites in Figure S7. The short answer is yes, functional SPSs are present in validated enhancers next to the down-regulated *Hes1* and *Hes5* gene. Though putative enhancers with SPS are located near *Dtx1* and E2F1, none are near *FoxM1*. The function of these enhancers in MZB needs to be elucidated, as no split Dam signal was detected in these regions in mK4 cells (image below presented here for the reviewers, we will only state this in the manuscript).

Minor comments

- in page 8, "1/8 surviving P0 N1+/-; N2+/- pups" -> "1/8 surviving P0 N1RA/-; N2RA/- pups

- The labels in Figure 5B are improperly presented (overlapped).

x

Thank you, we fixed these.

Hass, M.R., Liow, H.H., Chen, X., Sharma, A., Inoue, Y.U., Inoue, T., Reeb, A., Martens, A., Fulbright, M., Raju, S.*, et al.* (2015). SpDamID: Marking DNA Bound by Protein Complexes Identifies Notch-Dimer Responsive Enhancers. Mol Cell *59*, 685-697.
