# Supplementary material for: Notch dimerization and gene dosage are important for normal heart development, intestinal stem cell maintenance, and splenic marginal zone B-cell homeostasis during mite infestation
Source: PLoS Biol. 2020 Oct 5;18(10):e3000850. doi: 10.1371/journal.pbio.3000850 (PMC7561103; doi:10.1371/journal.pbio.3000850)
Supplement: Attachment — Submitted filename: Response to Academic Editor.docx [file pbio.3000850.s011.docx]

Academic Editor's comments

Dear Ines,

We addressed the comments, and corrected Figure 2H to reflect an error we found in calculating the significance of IL17A. We would like some guidance regarding supplemental Table S3- should it be mentioned once or after every figure? At present, we opted for the latter.

In Figure 1:

The N2RA has not been fully characterized and there is lack of evidence that N2RA would show similar dimerization deficiency like N1RA. It is recommended to point this out either here or in Discussion.

To address this fully we propose to add a panel (F) to Supplemental Figure S1. This panel shows that like Notch1, Notch2’s ability to activate Hes5 depends on dimerization. It also shows complementation between dimer mutants. The text was altered to reflect this data as follows:

To ask if loss of cooperativity impacts function *in vivo*, we used CRISPR-Cas9 to introduce amino acid substitutions at Arg^1974^ and Arg^1934^ in N1 and N2, respectively. Following the generation of a double strand break in Exon 32 of N1 or N2, a short oligonucleotide harboring two linked mutations was homologously recombined. Nucleotides coding for Arg^1974^ in N1 (N1R^1974^) and Arg^1934^ in N2 (N2R^1934^) were changed to code for Ala, creating the N1^R1974A^ (N1^RA^) and N2^R1934A^ (N2^RA^) mutations, respectively (Figure 1B, E). Dimerization-dependent activity of N2ICD and its ability to hetero-dimerize with N1ICD suggests it too binds cooperatively to SPS (Supplemental Figure S1F).

The legend in the supplemental figures has been extended to explain the data:

(F) Luciferase activation assays were used to analyze the homo and heterodimerization properties of Notch1 and Notch2 on a dimer-dependent reporter gene (Hes5-Luciferase) and a dimer-agnostic reporters (Hey2-Luciferase). As previously shown (Arnett et al., 2010), the dimer interface involves three amino acids, the Arg (R) mutated in this study, as well as the positively charged Lys (K1935, Notch1) and a negatively charged Glu (E1939, Notch1 or Asp (D1899) at the equivalent position in Notch2) that form salt bridges between NICD molecules. Dimer formation is critical for Notch-dependent activation of the Hes5 reporter, and mutating any of these amino acids abrogates activation. Notably, by co-expressing two Notch protein with complementary mutations, one in which K1935 was changed to E (N1KE), and a second in which D1899 was changed to K (N2DK) leads to strong enhancement in Luciferase expression, explained by achieving a more favorable conformation of the complementing mutant dimer. Note synergistic complementation between N1KE and N2DK, but not between N1KE and N2KD, provides strong evidence of a cooperative heterodimer. Significance tested by a student t test.

In Figure 7:

The overall analysis of this part is rather poor and may not qualify the level of requirement of our journal. I feel that the rest is already sufficient to confirm that dimerization deficiency of mammalian NotchICD can cause pathology in the gut, heart, and B cells. Therefore, I suggest to remove this part from the manuscript unless some of the issues below are addressed:

2. The RNAseq analysis with N=3 has failed in showing consistency within each group by having an outlier. Unless it has clear correlation to the severity of disease phenotype of the sacrificed mouse, it seems very difficult to make any conclusion here. Fig7D could be used to show the penetrance of increased expression of the selected genes – number of samples for each genotype is not shown and here many mice would need to be tested to make a firm conclusion.

This is a fair point; we removed the RNAseq data (Figure 7A-D) and modified the text accordingly.

3. Given the lack of solid evidence that Myb and FoxM1 are involved here, further chromosomal analysis with ATAC seq and SplitDAM (although I greatly appreciate the methods) doesn’t seem yield any solid conclusion. This all might need further in-depth study to reveal the true molecular mechanism of the observed phenotypes (for all three main phenotypes).

We agree that the ATAC data alone does not provide a mechanism, but we would like to keep it in a revised Figure 7 because it helps to eliminate altered chromatin near genes such as Myc, Myb and FoxM1 as a possible mechanism that contributes to the SMZL phenotype seen in N2RA animals. We could however remove it and the references made to it in the result section if the Academic Editor feels strongly about not including it.

The Fig S6A doesn’t look consistent among different replicates. S6B also show high level of variation, so it is extremely difficult to make any conclusion.

We again agree with the editor; the replicates indeed vary. Consider however that the consistent message is that we could not find evidence for stabilization. This is a representative result of multiple experiments done in tissue extracts from several organs.

We now state:

To test if the similarity of our phenotype to SMZL might in part be reflective of N2ICD stabilization, we assessed N2ICD levels in nuclear extracts from wild type and *N2^RA/RA^* MZB cells. While we see high variability between replicates, we find no evidence for a significant accumulation of nuclear N2ICD in sorted *N2^RA/RA^* MZBs (Supplementary Figure S6). These findings suggest that the gain-of-function phenotypes observed in *N2^RA/RA^* MZB cells are not due to enhanced N2ICD stabilization. Thus, unlike in many human SMZL patients, increased N2ICD stability is unlikely to be the cause of the SMZL phenotype in the *N2^RA/RA^* mice.

In conclusion, the suggestion would be to finish the manuscript at Fig 6 and Fig S5. Fig 8 might be included but after revising the contents. Perhaps a different schematic drawing to show dimerization deficiency mechanism and the affected pathological aspects in mouse would work better.

Since Figure 8 was always hypothetical, we modified it to indicate that the MZB mechanism was a speculation, based on the published roles of Myb and FoxM1 in promoting B cell proliferation.

Arnett, K.L., Hass, M., McArthur, D.G., Ilagan, M.X., Aster, J.C., Kopan, R., and Blacklow, S.C. (2010). Structural and mechanistic insights into cooperative assembly of dimeric Notch transcription complexes. Nat Struct Mol Biol *17*, 1312-1317.
